# Advanced machine learning model for better prediction accuracy of soil temperature at different depths

**DOI:** 10.1371/journal.pone.0231055

**Published:** 2020-04-14

**Authors:** Meysam Alizamir, Ozgur Kisi, Ali Najah Ahmed, Cihan Mert, Chow Ming Fai, Sungwon Kim, Nam Won Kim, Ahmed El-Shafie

**Affiliations:** 1 Department of Civil Engineering, Hamedan Branch, Islamic Azad University, Hamedan, Iran; 2 Department of Civil Engineering, Ilia State University, Tbilisi, Georgia; 3 Institute of Energy Infrastructure (IEI), Universiti Tenaga Nasional, Kajang, Selangor, Malaysia; 4 Faculty of Computer Technologies and Engineering, International Black Sea University, Tbilisi, Georgia; 5 Department of Civil Engineering, College of Engineering, Universiti Tenaga Nasional, Kajang, Selangor, Malaysia; 6 Department of Railroad Construction and Safety Engineering, Dongyang University, Yeongju, Republic of Korea; 7 Department of Land, Water and Environment Research, Korea Institute of Civil Engineering and Building Technology, Goyang-daero, Ilsanseo-gu, Goyang-si, Gyeonggi-do, Republic of Korea; 8 Department of Civil Engineering, Faculty of Engineering, University Malaya, Kuala Lumpur, Malaysia; 9 National Water Center, United Arab Emirates University, Al Ain, United Arab Emirates; University of Rochester, UNITED STATES

## Abstract

Soil temperature has a vital importance in biological, physical and chemical processes of terrestrial ecosystem and its modeling at different depths is very important for land-atmosphere interactions. The study compares four machine learning techniques, extreme learning machine (ELM), artificial neural networks (ANN), classification and regression trees (CART) and group method of data handling (GMDH) in estimating monthly soil temperatures at four different depths. Various combinations of climatic variables are utilized as input to the developed models. The models’ outcomes are also compared with multi-linear regression based on Nash-Sutcliffe efficiency, root mean square error, and coefficient of determination statistics. ELM is found to be generally performs better than the other four alternatives in estimating soil temperatures. A decrease in performance of the models is observed by an increase in soil depth. It is found that soil temperatures at three depths (5, 10 and 50 cm) could be mapped utilizing only air temperature data as input while solar radiation and wind speed information are also required for estimating soil temperature at the depth of 100 cm.

## Introduction

For different climatic zones whether it is tropical, arid or semi-arid, soil temperature is considered as one of the most essential variables affection the agricultural water management and process. As a result, forecasting the soil temperature could be of importance for water resources planners, especially for agricultural water demand. The fluctuations of the soil temperature at different time-increments hourly, daily or monthly plays a substantial role on the moisture status of the soil at different depth and governs the exchange of the energy and moisture in the boundary of the soil-atmosphere interaction layers [1]. In general, the soil temperature is the key factor for the successfulness of the agricultural process as it dominates the evaporation and the evapotranspiration, plant growth, ventilation and root conditions [2,3]. Furthermore, the soil temperature influences the status of the microorganisms and its activities within the soil (reference). The fact that the soil temperature alters with respect to the depth (keep in mind that the fluctuation of the temperature at the soil surface is higher than in deeper level) motivates the researcher to recommend the necessity for monitoring the soil temperature at different depth. In this context, it is necessary to monitor and evaluate the soil temperature at different depths [4,5].

In fact, the basic factor affecting the soil temperature and its distribution within the soil depth the climatic variables including air temperature, relative humidity, wind speed, solar radiation, rainfall, atmospherics and sunshine duration. Generally, most of the existing studies on soil temperature relied on a few or all these variables to predict the soil temperature [6,7,8]. It should be noted that in few cases most of these variables might not available and the interrelationship between the soil temperature and these variables are highly non-linear. Such truth, the ability of the machine learning models motivates the researchers to be utilized as the most effective technique to accurately predict the soil temperature [9,10,11].

During the last two decades, the machine learning methods have been applied and showed high effectiveness and accurate performance to several engineering applications, especially for forecasting, prediction, pattern recognition problems. In 2014, Coactive Neuro-Fuzzy Inference System (CANFIS) has been employed to forecast the daily soil temperature in arid and semi-arid areas by [12]. Relatively good performance for forecasting the soil temperature has been achieved, however, the range of the maximum error was slightly high. For the Bandar Abbas and Kerman stations in Iran, Nahvi et al. [13] developed modified version of the Extreme Learning Machine (ELM) by integrating with Self-adaptive Evolutionary (SaE) algorithm and introduced (SaE-ELM) model. The model has been structured considering the atmospheric pressure, air temperature and global solar radiation as inputs. It has been verified that the soil temperature forecasting accuracy has been slightly improved using the SaE-ELM model.

Furthermore, in 2017, Adaptive Neuro-Fuzzy Inference System (ANFIS), Gene Expression Programming (GEP), and Artificial Neural Network (ANN) methods have been utilized as a modeling technique for estimating the soil temperature (ST) at various depths for two different stations in Turkey by [11]. It has been reported that the GEP method outperformed the other methods attaining better accuracy for forecasting the soil temperature at all depths. In the same year, Mehdizadeh et al. [14] examined the GEP as a forecasting model for monthly soil temperatures of 31 stations in Iran. However, the model has been developed using different set of input variables including geographical information and period component rather than relied on the traditional meteorological variables as reported earlier. The achieved results from the study showed that the utilization of the ANFIS model enhanced the prediction accuracy of the soil temperature for all the 31 stations.

It has been reported that the major challenges for achieving accurate predicting of the soil temperature is unavailability of the most meteorological variables needed as the model inputs [12]. In addition, the prediction of the soil temperature has inner uncertainties in terms of the measurement sensors’ precision, a noise because of sensors and the nonlinear feature interrelationship. The conventional forecasting/predicting methods are found to be inappropriate for meeting these requirements specifically when it is required for forecasting the nonlinear dynamical variable in nature. Additionally, it is difficult to implement the classical modelling techniques when the system behavior is anonymous or slightly known. In this case, the use of new techniques becomes very essential in especially such a complex nonlinear dynamical system. In addition, for forecasting applications that includes several inputs, the selections of the most appropriate input selections are considered the most significant step in developing the forecasting model. Therefore, the selection of the minimum possible parameters that enclosed the most essential information for the model to be able to accurately forecast the desired parameter is vital step in structuring the model. In our study for forecasting the soil temperature, there are several inputs that should be considered in the model and it might be necessary to use different combinations of parameters due to the unavailability of some parameters. The model’s input selection is a necessary step to assure the successfulness of the model performance achieving accurate prediction accuracy for the model’s output. However, the existing research manuscripts for ST prediction did pay attention for this step as long as the required data are available for the model developers. On the other hand, the availability of the model’ inputs variables are not necessarily accessible for all case studies. Therefore, in this study, there is a need to investigate the potential for developing accurate ST prediction model relying on the most suitable model’s input pattern. In this context, it will be curious to introduce a method that might able to automatically prior select the most appropriate input selections. In this context, Group Method of Data Handling (GMDH) method has been employed in order to optimally select the appropriate input parameters for soil temperature at different depth [15]. GMDH is considered as an effective self-organizing algorithm that able to be adapted with machine learning method and permits the accomplishment of proper selection from database.

It should be noted that in order to acquire accurate ST values in the field that there is a need install several thermometers at several soil depths. In addition, the installation should be carried out at different locations within the study area at the same time to assure the consistency and the accuracy of the collected data [16,17]. The implementation of these procedures several are definitely costly and time-consuming especially in developing countries [18]. As a result, the accessibility of accurate and consistent ST data are very limited and hence there a need for robust model that able to capture the mapping between the input(s) and the ST as the model’s output Feng et al. [19]. Recently, Mehdizadeh et al. [20] developed Fractionally Autoregressive Integrated Moving Average (FARIMA) model so as to predict the ST and compare the results with classical Artificial Intelligent (AI) models namely; Gene Expression Programming (GEP) and Feed Forward Back Propagation Neural Network (FFBPNN) methods. Although that the results showed that FARIMA outperformed the FFBPNN and GEP methods, the prediction accuracy for ST using FARIMA were relatively inadequate for the extreme ST values, Mehdizadeh et al. [20].

Due to the highly expensive costs and the extensive delinquent and difficulty for direct measurements of soil temperature which is essential for several applications in meteorological, hydrological and agricultural process, it becomes crucial to examine the potential of machine learning methods to estimate the soil temperature. In this context, the current study, an investigation for predicting the soil temperature utilizing several machine learning methods has been proposed and assessed. As it has been reported earlier, it could be noticed that there were a lot of research efforts have been developed to predict the ST at different depths. However, the major inadequacy that have been experienced upon utilizing those models in predicting the ST, using the traditional statistical model such as ARIMA, is that there is a need to predefine proper stochastic procedure to identify the associated uncertainty for all used variables in the model. In addition, for the recently classical machine learning models, prior interrelationship information between different used variables, for example, covariance, variance and correlation values have to be accurately recognized to select the proper model’ input-output architecture. Furthermore, the classical machine learning models experienced over-fitting problems that could lead to unexpected relatively high prediction errors when different input patterns are examined. In this study, in fact, the ELM method has been developed in order to bridge the research gaps and drawbacks in these prediction modeling methods. The ELM has been proved to be reliable procedure and promising algorithm for overcoming the over-fitting problems. In fact, the ELM’s procedure is designed to overcome the disadvantages of both prediction modeling concepts, the traditional and machine learning. The ELM’s procedure allows to considerably minimize the possibility of experiencing over-fitting while training and hence consistent prediction accuracy for unexpected input pattern could be achieved. In addition, the random projection procedure within parallel computing techniques increase the possibility of accomplishing successful convergence procedure and lessen the time needed to achieve the performance goal.

The purpose of the model is to predict the soil temperature at different depths (5, 10, 50 and 100cm) on a monthly basis. So as to substantiate the exactness of the developed methods, comprehensive comparative analysis has been carried between the proposed machine learning models including CART, GMDH, ELM, and ANN. In addition, different grouping and pattern of climate variables have been examined as inputs for the model including air temperature, solar radiation, relative humidity and wind speed. Different statistical indices have been evaluated to examine the performance of the models to compare how accurate is the model output with the desired soil temperature at different depths. It should be noticed here that it is the first attempt to utilize both the CART and GMDH models as a predictor for soil temperature.

## Materials and methods

### Used data

**Fig 1:**
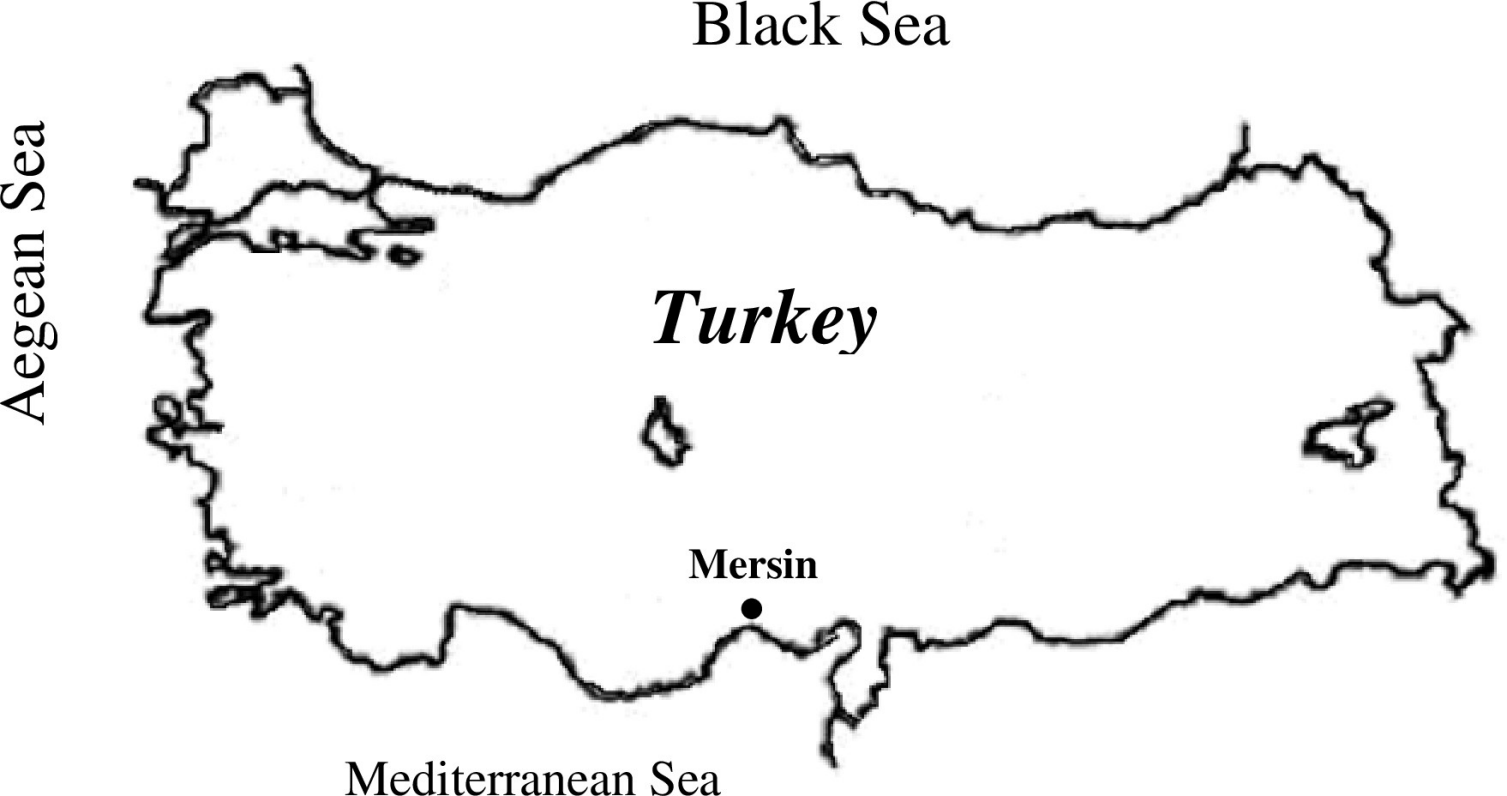
The location of the station in Mediterranean region of Turkey.

In the study, monthly climatic data, air temperature (T), relative humidity (RH), solar radiation (SR), wind speed (W) and soil temperature for the depths of 5, 10, 50, and 100 cm were obtained from Mersin station (longitude 34° 38′ E, latitude 36° 48′ N, altitude 3 m) which is operated by Turkish Meteorological Service. The study area (Fig 1) has Mediterranean climate with wet winters and dry summers [21,11]. The winter can get very heavy rains and flooding is a big problem in some regions. The air temperature ranges from 24°C (winter) to 40°C (summer). The data cover 25-year monthly records from 1986 to 2010. In the study, the first 80% was utilized for training and remaining 20% was utilized for testing.

**Fig 2:**
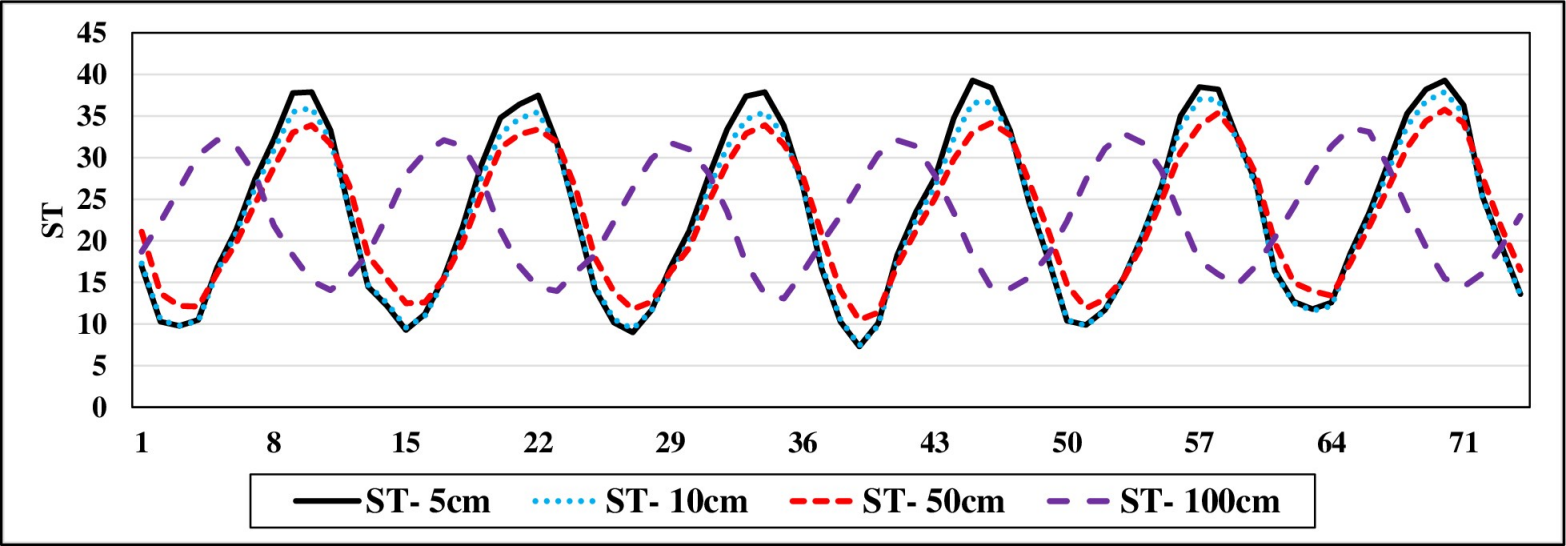
Soil temperature variation at 5cm, 10cm, 50cm, and 100cm.

**Fig 3:**
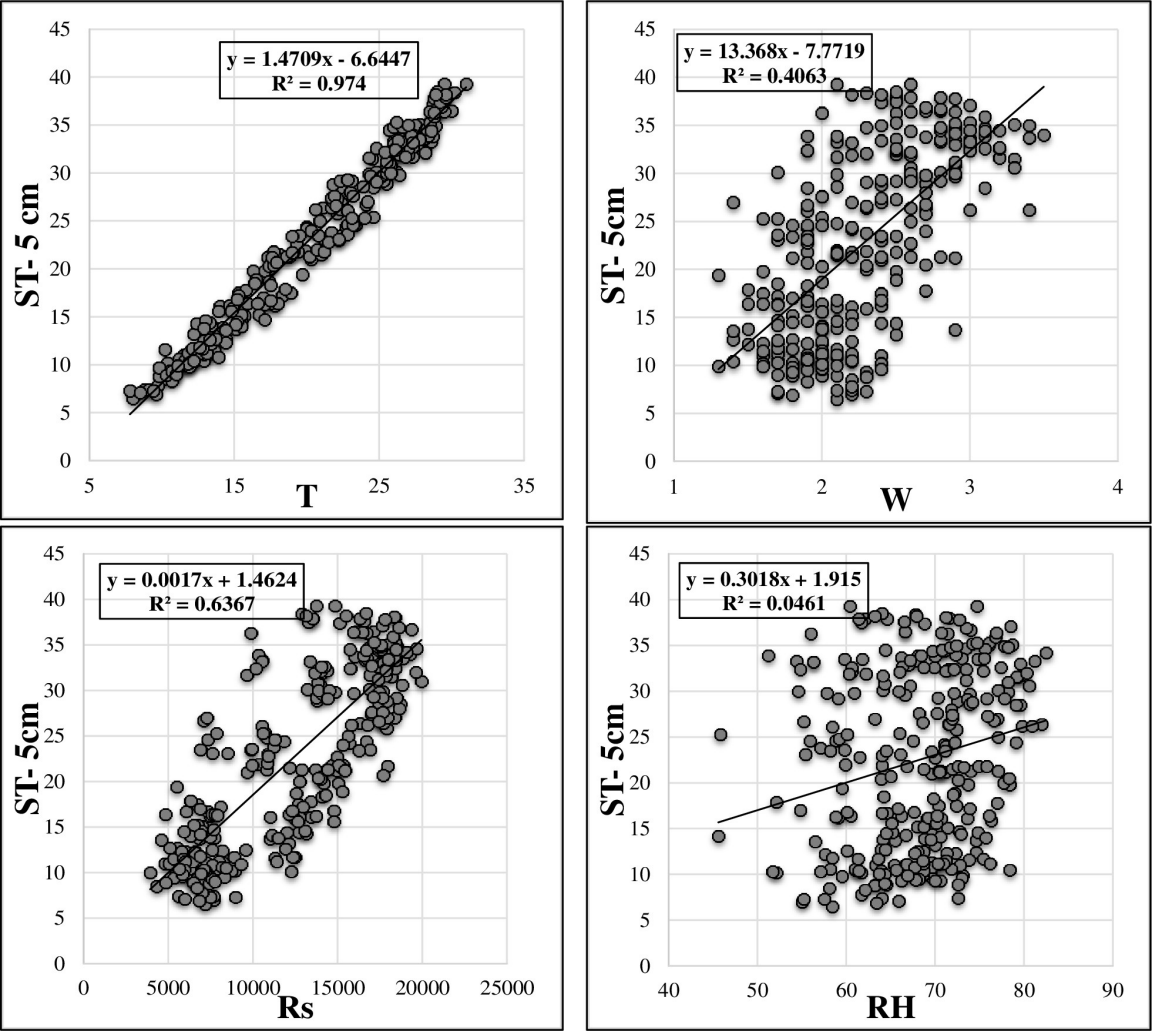
Relation between input parameters and ST- 5cm.

**Fig 4:**
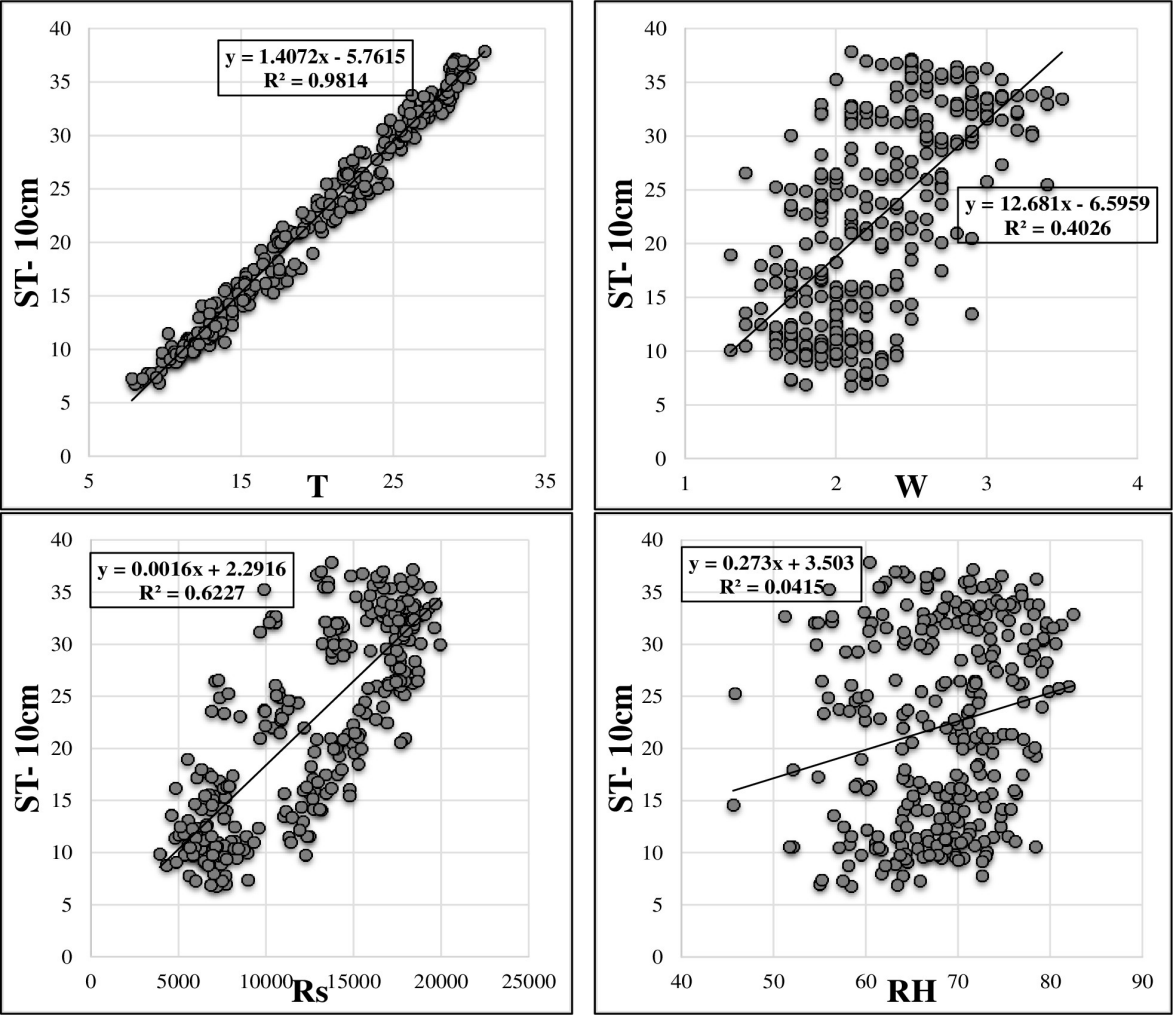
Relation between input parameters and ST- 10cm.

**Fig 5:**
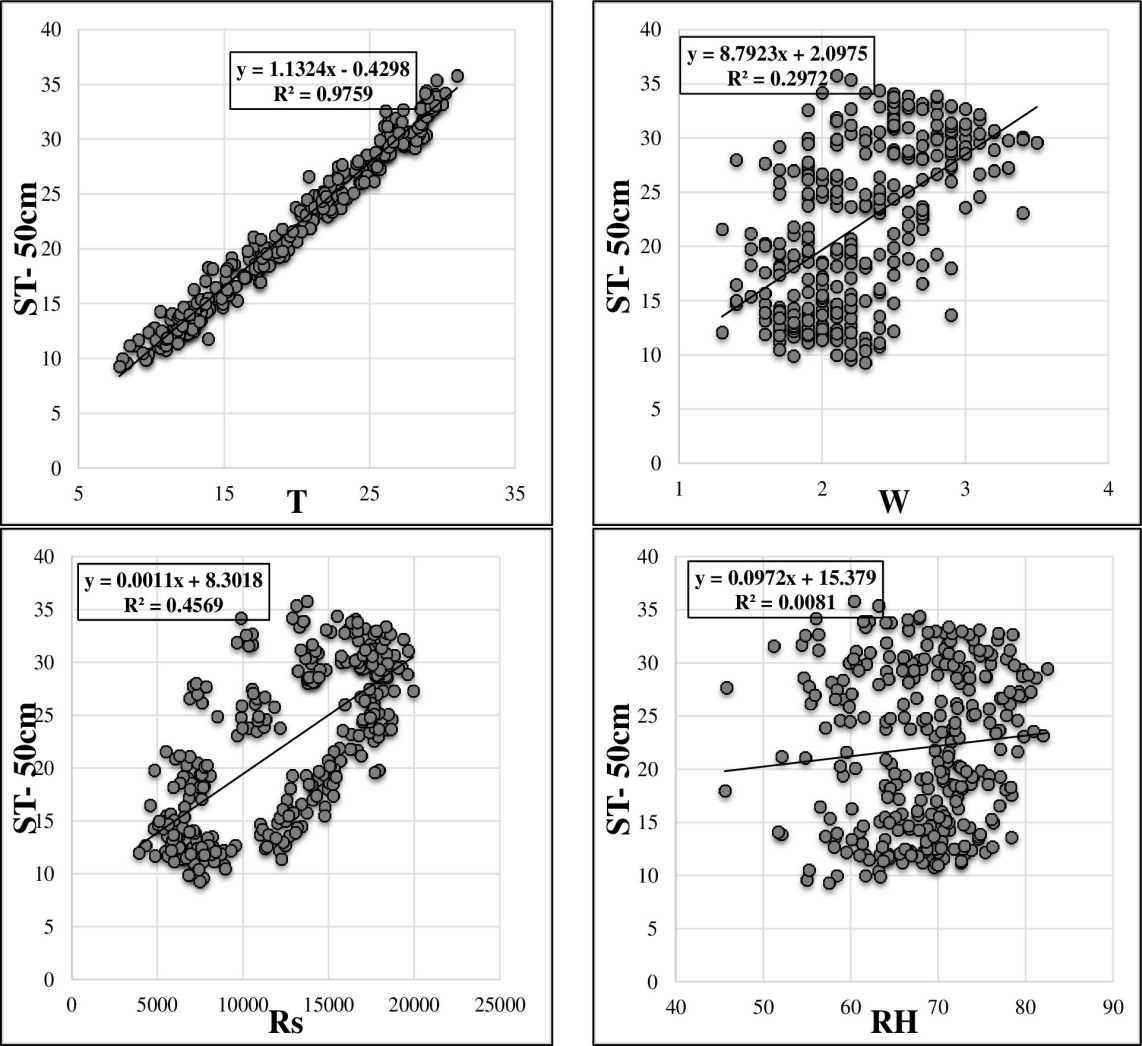
Relation between input parameters and ST- 50cm.

**Fig 6:**
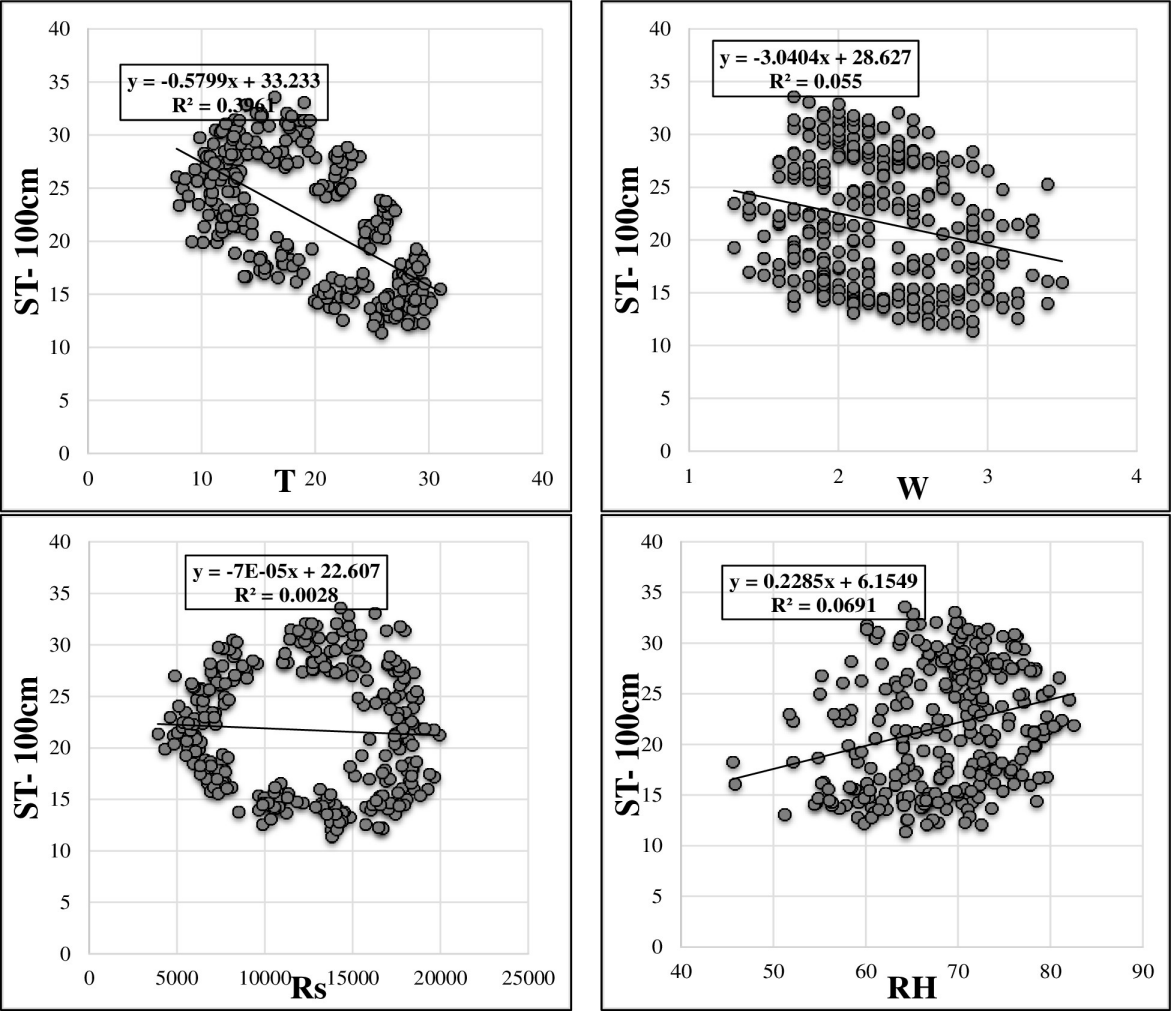
Relation between input parameters and ST- 100cm.

Table 1 sums up the brief statistical properties of the climatic and soil temperature data. It is apparent that ST data show normal distribution (skewness values are close to 0). Maximum values of the soil temperatures at different depths are higher in the test phase in comparison with training phase. This may limit the extrapolation capabilities of the implemented models [22,23]. It can be said that the variation of ST decreases with respect to depth increment (see St. Dev. in Table 1). The soil temperature variation at various depths is illustrated in Fig 2. As observed, ST at the depths of 5 cm, 10 cm and 50 cm have high correlations while the ST at 100 cm has totally different variation compared to other values of three depths. Figs 3–6 demonstrate the visual relationships between each climatic input and ST values for different depths. It is clear that the air temperature is highly correlated with ST especially for the first three depths and it is followed by the Rs, W and RH, respectively.

**Table 1:** Brief statistical parameters of the soil temperature and climatic data.

	Data set	Unit	Avr.	Min.	Max.	St. Dev.	Skewness
Training data	T	°C	19.6	7.8	30	6.52	-0.04
	Rs	cal/cm^2^	12373	3921	19941	4599	-0.11
	W	m/sec	2.33	1.3	3.5	0.46	0.34
	R_H_	%	69.3	54.4	82.5	6.28	-0.26
	ST-5cm	°C	22.1	6.5	38.1	9.58	0.02
	ST-10cm	°C	21.8	6.8	37.2	9.18	0.0005
	ST-50cm	°C	21.6	9.3	34.1	7.31	-0.0003
	ST-100cm	°C	21.4	11.4	32.1	5.82	0.06
Testing data	T	°C	20.7	9.4	31	6.54	-0.05
	Rs	cal/cm^2^	12061	4590	17989	4358	-0.18
	W	m/sec	1.99	1.3	2.8	0.37	0.15
	R_H_	%	63.8	45.6	78.2	7.61	-0.34
	ST-5cm	°C	24.1	7.3	39.3	10.3	0.01
	ST-10cm	°C	23.4	7.4	37.9	9.61	-0.01
	ST-50cm	°C	23.5	10.5	35.8	8.02	-0.03
	ST-100cm	°C	23.1	13.1	33.6	6.59	0.06

### Used methods

#### ANN

**Fig 7:**
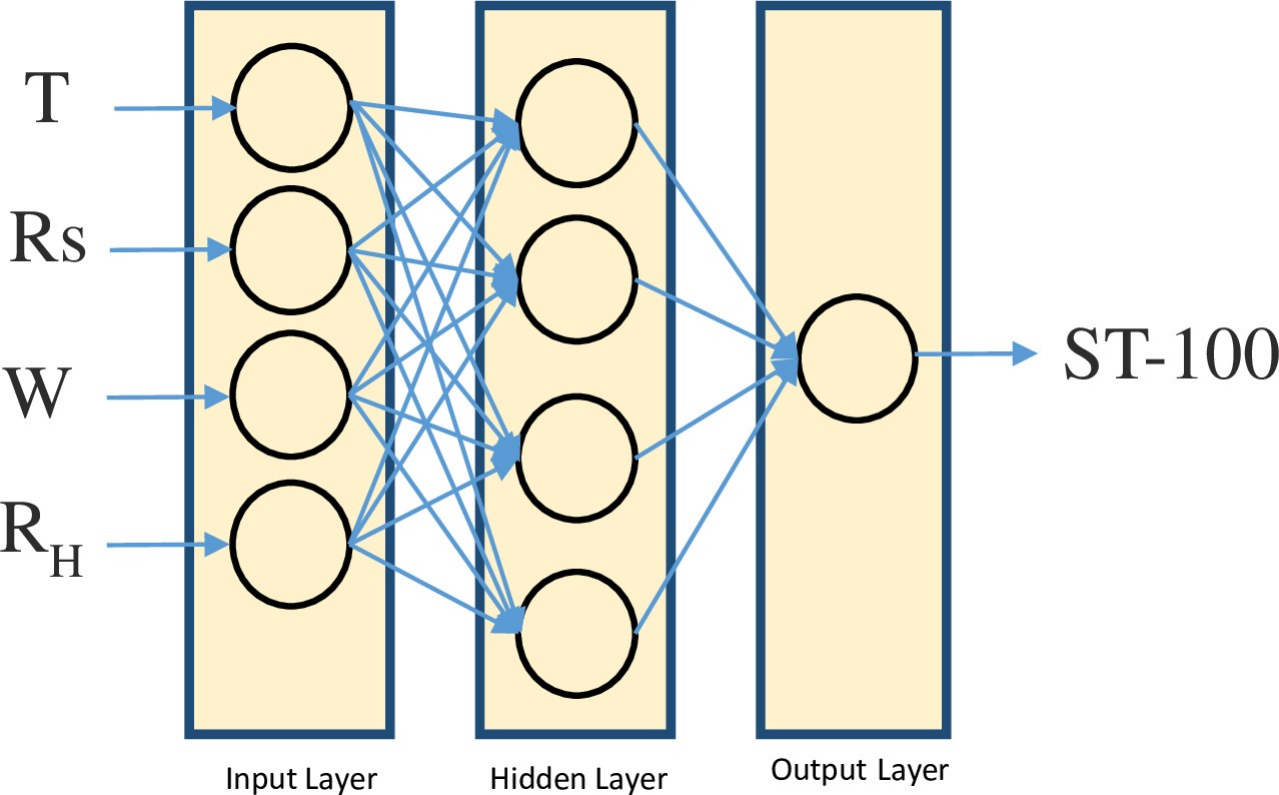
Architecture of an artificial neural network.

Artificial neural network (ANN) is a data processing based on the neural structure of the human brain. It constructs relations between inputs and outputs. It has parallel data processing architecture like human neural system [24]. The basic element of a human neural system is a neuron, which has four basic components. Neurons receive weighted inputs, combine them, apply nonlinear operation and give the output. Therefore, the artificial neuron, which is the elementary processing element of an ANN, has four functions like natural neurons. Clustering these artificial neurons forms the artificial neural network. This clustering happens by making layers, which are then associated with each other. Most applications need ANN to have three interconnected layers, which are input, hidden and output layers. Fig 7 illustrates an ANN architecture with three inputs, four hidden nodes and two outputs. These ANNs are known as multi-layer perceptron (MLP) [25,26,27]. The connection among layers is the one of the most important features of an ANN. It can be feedforward or feedback. Feedforward networks are unidirectional while feedback networks have loops.

One of the most recognized advantages of an ANN is that it can learn. Learning occurs by adjusting weights to minimize the error between predicted and observed values. There are different training algorithms, which minimizes the error. One of the most popular training algorithms is Bayesian regularization, which is also used in this study [28]. The following formula shows the sigmoid function which is used in this study.

$$S(x)=\frac{e^{x}}{e^{x}+1}$$ (1)

Learning occurs by adjusting weights to minimize the error between predicted and observed values. There are different training algorithms, which minimizes the error. One of the most popular training algorithms is Bayesian regularization, which is also used in this study [28].

Besides theoretical complexity and needs of fine tuning weakness of ANN, one of the main advantages of this method is its effectiveness in complex relational variables and high dimensional problems.

#### CART

**Fig 8:**
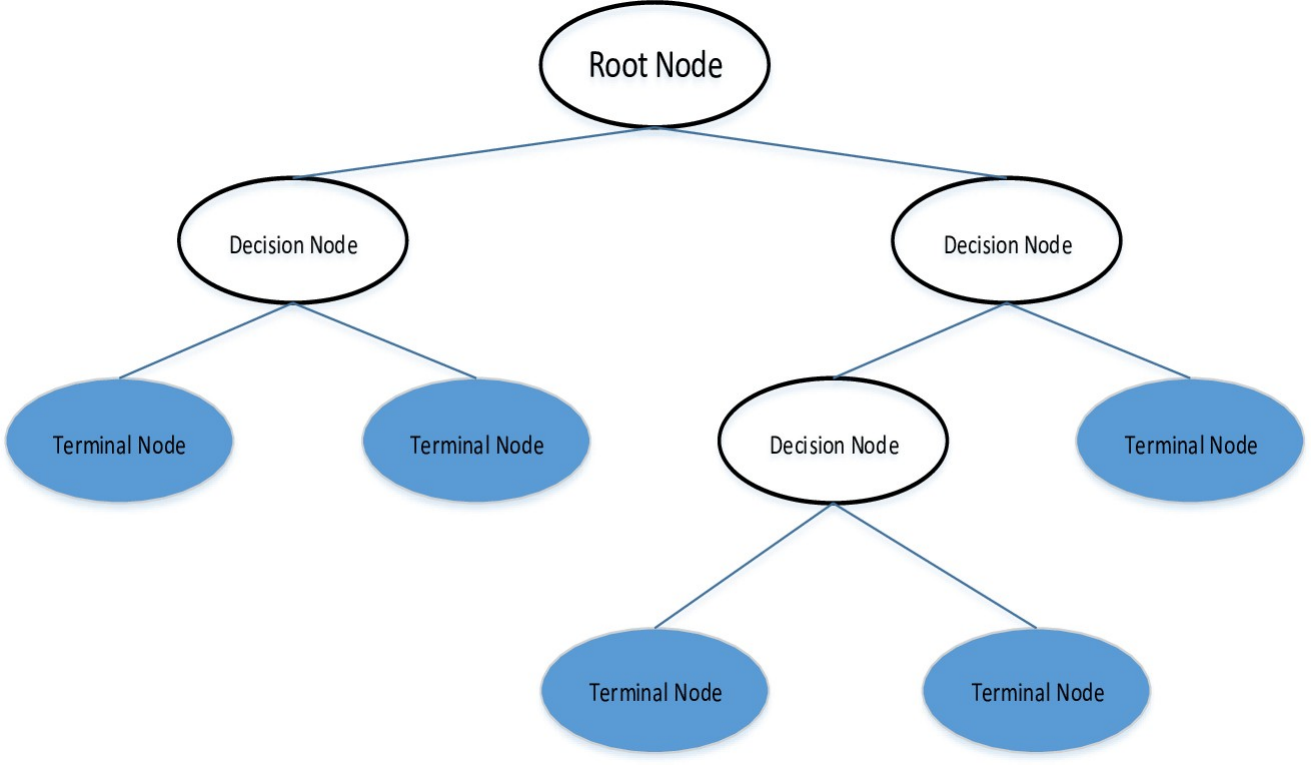
Decision tree.

Leo Breiman et al. [29] introduced the classification and regression tree (CART). It is used as a prediction model, which uses a binary decision tree, see Fig 8 [30,31,32]. It is specially fitted for tasks in which a little priori knowledge exists. The motivation behind the analyses by means of tree-building algorithms is to decide split conditions, which gives correct classification of cases or prediction. CART divides the datasets into child nodes until it reaches stable state in which dividing leaf nodes don’t improve the entire tree. There are three main steps in the CART methodology. These steps are tree growing, tree pruning, and selection of optimal tree. Since CART integrates continuous and categorical dependent variables, it is widely used by many practitioners.

Friedl et al. [33] noted that boosting and bagging algorithms can improve the performance of CART algorithm. Deciding about a threshold value and input feature are the important parts of the forming the structure of the decision tree. Since it is decision tree it naturally discards the ineffective input features. CART models are very interpretable since the impact of each input variable on the output can be envisioned by the related tree-based structure.

#### ELM

For the past decades in the feed forward neural networks the learning speed was generally slower than desired since the entire network parameters are iteratively tuned mostly using slow gradient-based learning algorithm. Also, more human involvement is needed in classical learning methods to get suitable model parameters. Extreme learning machine (ELM) is proposed as a new machine learning method to get the better of the classical machine learning models for single-hidden layer feed-forward neural networks (SLFNs) [34,35]. It simply uses the idea of a random projection and then linear regression. During its learning phase tuning hidden neurons is not required. A sub-network of several nodes can be used as a hidden node.

In the last decades it has been used in variety problems like feature learning, classification, regression, and clustering [36,37,38,39,40,41,42,43]. ELM became faster and efficient for big data processing because of the improvements of the parallel computing techniques.

The output function of generalized single- hidden layer feed-forward neural networks with L hidden nodes can be defined as:

$$f_{M}(x)=\sum_{\left(i=1\right)}^{L}\beta_{i}G(a_{i},b_{i},x)$$ (2)

where β_1_ is output weights and G is hidden node output function.

During training phase, the quantity of hidden nodes, output function and hidden node is given, ELM learning process consists of the following steps:

Assigning randomly hidden node parameters (*a*_*i*_,*b*_*i*_), i = 1,…,L, calculating hidden layer output matrix:

$$H=[\begin{array}{ccc} G(a_{1},b_{1},x_{1}) & \cdots & G(a_{L},b_{L},x_{1})\\ \vdots & \ddots & \vdots \\ G(a_{1},b_{1},x_{N}) & \cdots & G(a_{L},b_{L},x_{N}) \end{array}],$$ (3)

and calculating output weights *β* by minimizing ‖*Hβ*−*T*‖ and ‖*β*‖ where $T=\left[\begin{array}{c} t_{1}^{T}\\ \vdots \\ t_{N}^{T} \end{array}\right]$ is the target function [34].

#### GMDH

Group method of data handling (GMDH) is originated by Ivakhnenko [44]. It is a set of inductive algorithms. It consists of clusterization, rebinarization, parametric, probability and analogues complexing algorithms. It has big area of applications like optimization, data mining, complex system modeling, pattern recognition, and deep learning. It is cited as one of the oldest deep learning methods [45]. By its inductive nature it finds the optimal structure of the model without interaction of the authors.

**Fig 9:**
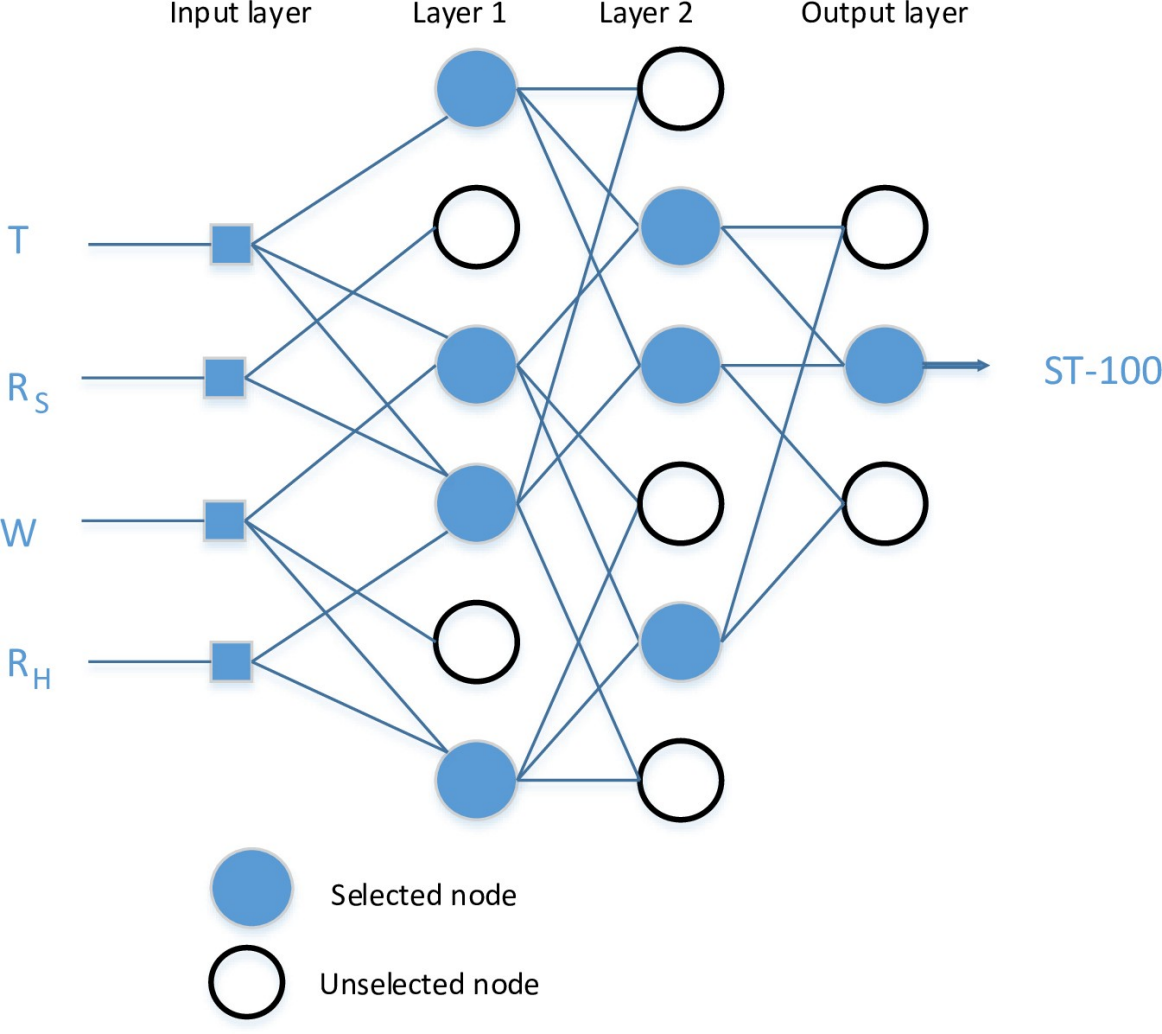
A typical GMDH neural network.

The performance of GMDH is better than the classical alternatives like ARIMA, back-propagation neural network, single exponential smooth [46]. GMDH has three parts which are input variables, external and internal criteria, see Fig 9. During model selection, external criteria are used meanwhile internal criteria are used to predict the equation’s coefficients.

Polynomial functions are used in the majority of GMDH algorithms and the network formed by using GDMH is adaptive. A generic relation between input/output variables can be shown by the following Kolmogorov- Gabor polynomial equation [47].

$$y=a_{0}+\sum_{i=1}^{m}a_{i}x_{i}+\sum_{i=1}^{m}\sum_{j=1}^{m}a_{ij}x_{i}x_{j}+\sum_{i=1}^{m}\sum_{j=1}^{m}\sum_{k=1}^{m}a_{ijk}x_{i}x_{j}x_{k}+\cdots$$ (4)

where y is the node output, *x*_*i*_,*x*_*j*_,*x*_*k*_,… are inputs, and *a*_0_,*a*_*i*_,*a*_*ij*_,… are the coefficients of the polynomials.

## Application and results

In the presented work, the exactness of four data-driven methods, ELM, ANN, CART, GMDH and multi-linear regression (MLR), is examined in mapping monthly soil temperature at various depths. Sigmoid function and 250 hidden nodes were used for the ELM after trying various numbers. For the ANN models, Bayesian regulation was employed for training and the optimal hidden node number was determined as 25. The hidden neurons’ number was decided based on trial and error considering minimization of the training error and the performance of the models was checked by testing samples that had not any role in model training (calibration stage). In the hidden/output layers, sigmoid/purelin activation functions were utilized, respectively. The loss function was mean-squared error. Root mean square error (RMSE) was utilized to assess the employed models. To check the over training which is the well-known challenge in AI-based techniques, dataset was classified as training and testing and models were calibrated utilizing training samples and the performance of the models was checked by testing samples that had not any role in model training (calibration stage). Therefore, an acceptable result in testing stage proved that there is no over training in the proposed models. The following criteria were used for evaluation of the model results

$$Root\;Mean\;Square\;Error\;(RMSE)=\sqrt{\frac{\sum_{i=1}^{N}{\left({ST}_{im}-{ST}_{ip}\right)}^{2}}{N}}$$ (5)

$$Nash-Sutcliffe\;Efficiency\;(NSE)=1-\frac{\sum_{i=1}^{N}{\left({ST}_{im}-{ST}_{ip}\right)}^{2}}{\sum_{i=1}^{N}{\left({ST}_{im}-{\bar{ST}}_{m}\right)}^{2}}$$ (6)

$$Determination\;Coefficient\;(R^{2})=\frac{\sum_{i=1}^{N}{\left({ST}_{im}-{\bar{ST}}_{m})({ST}_{ip}-{\bar{ST}}_{p}\right)}^{2}}{\sum_{i=1}^{N}{\left({ST}_{im}-{\bar{ST}}_{m}\right)}^{2}\sum_{i=1}^{N}{\left({ST}_{ip}-{\bar{ST}}_{p}\right)}^{2}}$$ (7)

Where *N* = data quantity, *ST*_im_ = measured soil temperature, ${\bar{ST}}_{m}$ = mean of measured soil temperature, *ST*_ip_ = predicted soil temperature, ${\bar{ST}}_{p}$ = mean of predicted soil temperature.

Training and test results of the ELM models with various input scenarios are summed up in Table 2. At the first the depths of 5, 10 and 50 cm, the models with only temperature input performs superior to the other models while for the depth 100 cm, the ELM model with T, Rs, W or full inputs has the least RMSE and the highest NSE and R^2^. The best models’ accuracies of the ELM with respect to RMSE range from 1.190 cm (10 cm depth) to 3.211 cm (100 cm depth) in modeling soil temperature at various depths. Table 3 reports the training and test statistics of the ANN with different climatic inputs in estimation of soil temperature at 5, 10, 50 and 100 cm depths. The optimal ANN model was found for only temperature input for each depth. The RMSE range of the best ANN models is 1.429–5.407 cm (10 cm—100 cm depths).

**Table 2:** Training and testing accuracies of the ELM models in modeling soil temperature at different depths.

Input combination	Training			Testing		
	RMSE (°C)	NSE	R^2^	RMSE (°C)	NSE	R^2^
5 cm						
T	**1.526**	**0.974**	**0.974**	**1.607**	**0.975**	**0.980**
T, R_H_	8.531	0.196	0.727	10.08	0.033	0.335
T, Rs, W	8.131	0.269	0.744	9.705	0.103	0.337
T, Rs, W, R_H_	3.836	0.837	0.837	6.711	0.571	0.571
10 cm						
T	**1.265**	**0.980**	**0.980**	**1.190**	**0.984**	**0.986**
T, R_H_	8.129	0.210	0.708	9.339	0.048	0.312
T, Rs, W	7.663	0.298	0.762	8.693	0.175	0.312
T, Rs, W, R_H_	4.167	0.792	0.792	7.110	0.448	0.461
50 cm						
T	**1.025**	**0.980**	**0.980**	**1.397**	**0.969**	**0.982**
T, R_H_	6.245	0.263	0.572	7.875	0.017	0.181
T, Rs, W	5.949	0.331	0.607	7.411	0.13	0.177
T, Rs, W, R_H_	4.242	0.660	0.66	6.335	0.364	0.39
100 cm						
T	4.276	0.455	0.455	5.149	0.356	0.459
T, R_H_	5.492	0.102	0.102	7.201	-0.258	0.346
T, Rs, W	**2.210**	**0.854**	**0.854**	**3.211**	**0.749**	**0.901**
T, Rs, W, R_H_	2.185	0.857	0.857	3.215	0.749	0.915

**Table 3:** Training and testing accuracies of the ANN models in modeling soil temperature at different depths.

Input combination	Training			Testing		
	RMSE (°C)	NSE	R^2^	RMSE (°C)	NSE	R^2^
5 cm						
T	**1.600**	**0.971**	**0.971**	**1.914**	**0.965**	**0.977**
T, R_H_	9.276	0.049	0.130	10.82	-0.113	0.027
T, Rs, W	9.379	0.028	0.028	10.28	-0.005	0.052
T, Rs, W, R_H_	8.411	0.218	0.723	10.06	0.037	0.336
10 cm						
T	**1.237**	**0.981**	**0.981**	**1.429**	**0.977**	**0.980**
T, R_H_	9.046	0.022	0.022	9.596	-0.004	0.004
T, Rs, W	7.968	0.241	0.729	9.085	0.099	0.314
T, Rs, W, R_H_	8.069	0.221	0.709	9.310	0.054	0.314
50 cm						
T	**1.058**	**0.978**	**0.978**	**1.456**	**0.966**	**0.984**
T, R_H_	7.152	0.034	0.501	8.088	-0.035	0.085
T, Rs, W	6.846	0.114	0.537	8.465	-0.134	0.179
T, Rs, W, R_H_	6.538	0.192	0.557	8.146	-0.050	0.181
100 cm						
T	**4.488**	**0.400**	**0.401**	**5.407**	**0.290**	**0.406**
T, R_H_	5.628	0.057	0.057	6.446	-0.008	0.115
T, Rs, W	4.949	0.270	0.270	6.053	0.110	0.205
T, Rs, W, R_H_	4.328	0.442	0.442	5.704	0.210	0.409

The RMSE, NSE and R^2^ statistics of the CART are provided in Table 4. A different trend is observed for this method compared to ELM and ANN. Linear structure of CART may be the reason of this. The best CART models for the depths 5, 10, 50 and 100 cm were obtained from the fourth, second, first and the third input combinations, respectively. The RMSE of the best model increases from 1.269 cm (10 cm depth) to 3.330 cm (100 cm depth). Table 5 presents the training and test results of the GMDH in respect of RMSE, NSE and R^2^ in mapping soil temperatures of various depths. For this method, temperature input provides the best performance for the depths of 50 cm and 100 cm while for the other two depths, the model with T and RH (second input combination) has the best accuracy. The error range of the GMDH with respect to RMSE varies from 1.165 cm (50 cm depth) to 4.486 cm (100 cm depth). The training and test results of the MLR are reported in Table 6 in mapping soil temperature at four different depths. The best MLR models for the depths 5, 10, 50 and 100 cm were obtained from the first, first, second and the third input combinations, respectively. The RMSE increment of the best models is from 0.997 cm (50 cm depth) to 4.045 cm (100 cm depth). As a linear model, MLR seems to be worse than the other linear structured CART model except the ST at 50 cm in which the MLR with T and R_H_ input performs superior to the other four methods in this case.

**Table 4:** Training and testing accuracies of the CART models in modeling soil temperature at different depths.

Input combination	Training			Testing		
	RMSE (°C)	NSE	R^2^	RMSE (°C)	NSE	R^2^
5 cm						
T	1.162	0.985	0.985	1.719	0.971	0.974
T, R_H_	0.600	0.996	0.996	1.665	0.973	0.977
T, Rs, W	0.563	0.996	0.996	1.674	0.973	0.975
T, Rs, W, R_H_	**0.571**	**0.996**	**0.996**	**1.591**	**0.975**	**0.977**
10 cm						
T	0.981	0.988	0.988	1.407	0.978	0.979
T, R_H_	**0.570**	**0.996**	**0.996**	**1.269**	**0.982**	**0.984**
T, Rs, W	0.549	0.996	0.996	1.401	0.978	0.98
T, Rs, W, R_H_	0.551	0.996	0.996	1.365	0.979	0.98
50 cm						
T	**0.853**	**0.986**	**0.986**	**1.382**	**0.969**	**0.980**
T, R_H_	0.636	0.992	0.992	1.552	0.962	0.976
T, Rs, W	0.667	0.991	0.991	1.542	0.962	0.975
T, Rs, W, R_H_	0.668	0.991	0.991	1.541	0.962	0.974
100 cm						
T	2.975	0.738	0.738	5.478	0.299	0.386
T, R_H_	1.217	0.956	0.956	3.576	0.701	0.799
T, Rs, W	**1.178**	**0.959**	**0.959**	**3.330**	**0.741**	**0.821**
T, Rs, W, R_H_	1.121	0.962	0.962	3.483	0.716	0.787

**Table 5:** Training and testing accuracies of the GMDH models in modeling soil temperature at different depths.

Input combination	Training			Testing		
	RMSE (°C)	NSE	R^2^	RMSE (°C)	NSE	R^2^
5 cm						
T	0.937	0.990	0.990	1.726	0.971	0.986
T, R_H_	**0.934**	**0.990**	**0.990**	**1.643**	**0.974**	**0.984**
T, Rs, W	0.908	0.991	0.991	1.757	0.970	0.986
T, Rs, W, R_H_	0.877	0.991	0.991	2.294	0.949	0.951
10 cm						
T	0.806	0.992	0.992	1.308	0.981	0.987
T, R_H_	**0.807**	**0.992**	**0.992**	**1.218**	**0.983**	**0.988**
T, Rs, W	0.762	0.993	0.993	1.399	0.978	0.978
T, Rs, W, R_H_	0.757	0.993	0.993	4.425	0.784	0.799
50 cm						
T	**0.978**	**0.982**	**0.982**	**1.165**	**0.978**	**0.978**
T, R_H_	0.980	0.982	0.982	1.423	0.968	0.97
T, Rs, W	0.948	0.983	0.983	3.253	0.833	0.851
T, Rs, W, R_H_	0.937	0.983	0.983	6.279	0.377	0.52
100 cm						
T	**2.479**	**0.818**	**0.819**	**4.486**	**0.529**	**0.923**
T, R_H_	2.484	0.817	0.818	5.092	0.394	0.916
T, Rs, W	2.359	0.835	0.835	7.635	-0.361	0.287
T, Rs, W, R_H_	2.242	0.851	0.851	4.733	0.476	0.931

**Table 6:** Training and testing accuracies of the MLR models in modeling soil temperature at different depths.

Input combination	Training			Testing		
	RMSE (°C)	NSE	R^2^	RMSE (°C)	NSE	R^2^
5 cm						
T	**1.486**	**0.975**	**0.975**	**1.652**	**0.973**	**0.978**
T, R_H_	0.855	0.992	0.992	1.908	0.964	0.985
T, Rs, W	0.846	0.992	0.992	1.821	0.968	0.988
T, Rs, W, R_H_	0.835	0.992	0.992	1.883	0.965	0.985
10 cm						
T	**1.235**	**0.981**	**0.981**	**1.252**	**0.982**	**0.983**
T, R_H_	0.720	0.993	0.993	1.419	0.977	0.986
T, Rs, W	0.713	0.994	0.994	1.424	0.977	0.986
T, Rs, W, R_H_	0.737	0.993	0.993	1.376	0.979	0.985
50 cm						
T	1.056	0.979	0.979	1.286	0.973	0.983
T, R_H_	**0.907**	**0.984**	**0.984**	**0.997**	**0.984**	**0.988**
T, Rs, W	0.964	0.982	0.982	1.089	0.981	0.988
T, Rs, W, R_H_	0.998	0.981	0.981	1.222	0.976	0.978
100 cm						
T	4.366	0.436	0.436	5.201	0.368	0.500
T, R_H_	2.285	0.845	0.845	4.337	0.560	0.922
T, Rs, W	**2.181**	**0.859**	**0.859**	**4.045**	**0.617**	**0.923**
T, Rs, W, R_H_	2.274	0.847	0.847	4.091	0.609	0.915

**Fig 10:**
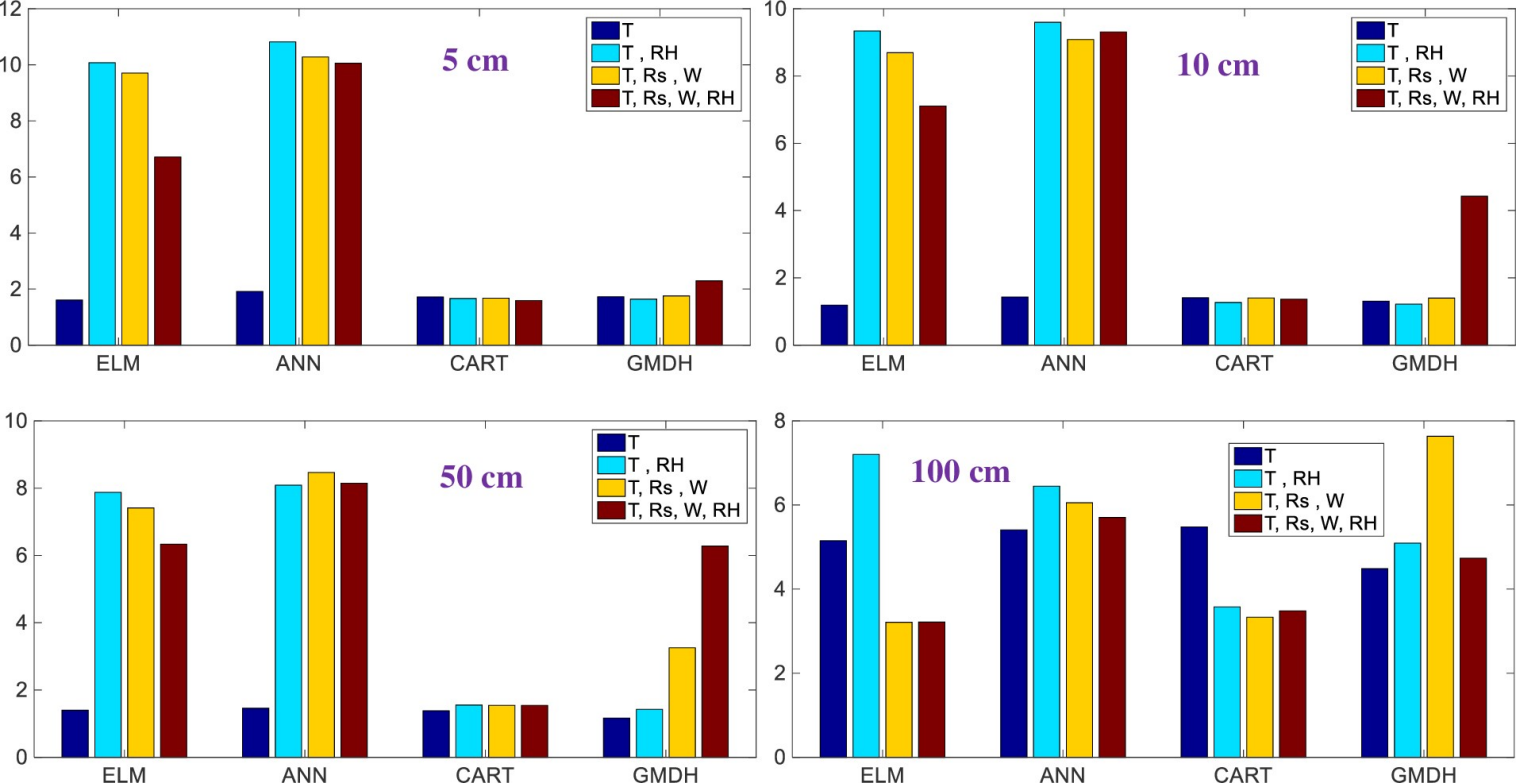
The RMSE values of the optimal ELM, ANN, CART, and GMDH models in predicting soil temperatures at different depths.

**Fig 11:**
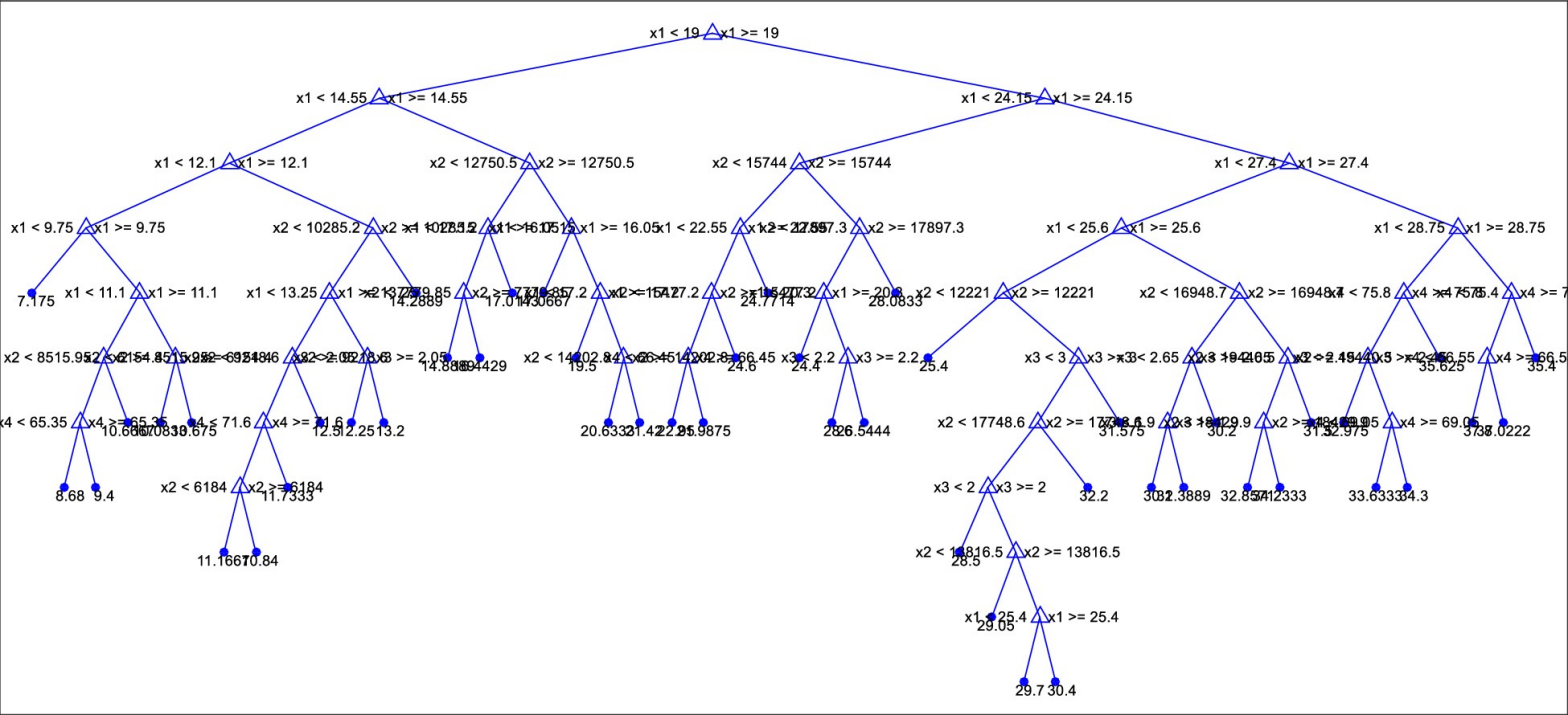
The tree of the optimal CART model in predicting soil temperature at 5-cm depth.

The RMSE values of the best models are visually compared in Fig 10 in the bar graph forms. The differences among the models with respect to various climatic inputs can be better seen from these graphs. In case of 5 cm depth, including climatic variables (RH, Rs and W) in inputs does not affect the accuracy of the CART and GMDH while the exactness of the ELM and ANN decreases and the one input model with temperature data has the lowest RMSE for the all methods. In estimation of soil temperature at 10 cm and 50 cm depths, also same trend is observed except the last one and two-input combinations of the GMDH. In case of 100 cm depth, adding Rs and W variables into input combination generally increases the performance of ELM and CART and they perform superior to the ANN and GMDH. As an example, the regression tree of the CART model for modeling soil temperature at depth of 5 cm is illustrated in Fig 11.

**Fig 12:**
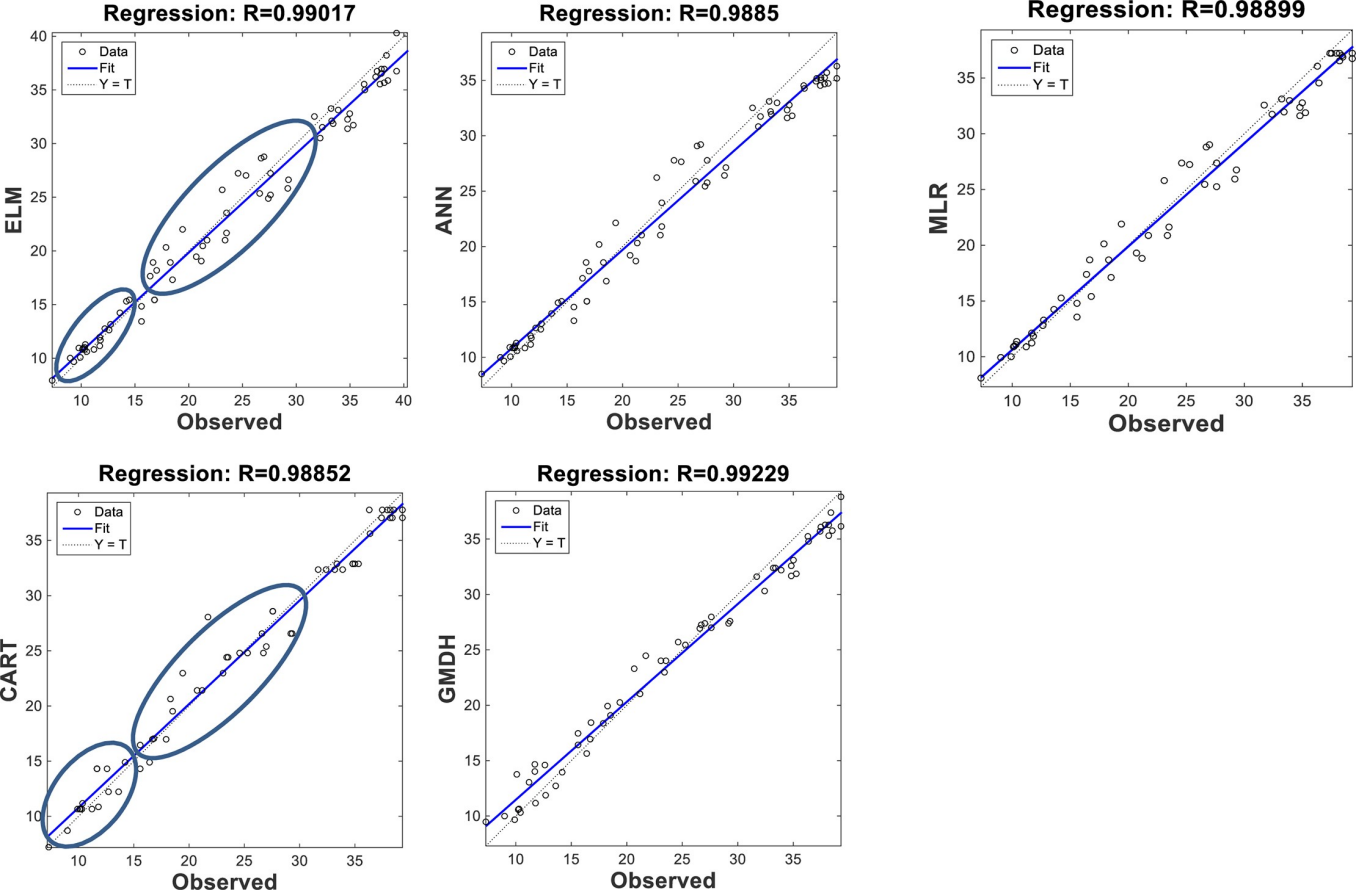
The scatterplots of the optimal ELM, ANN, CART, GMDH, and MLR models in predicting soil temperatures at 5 cm depth.

**Fig 13:**
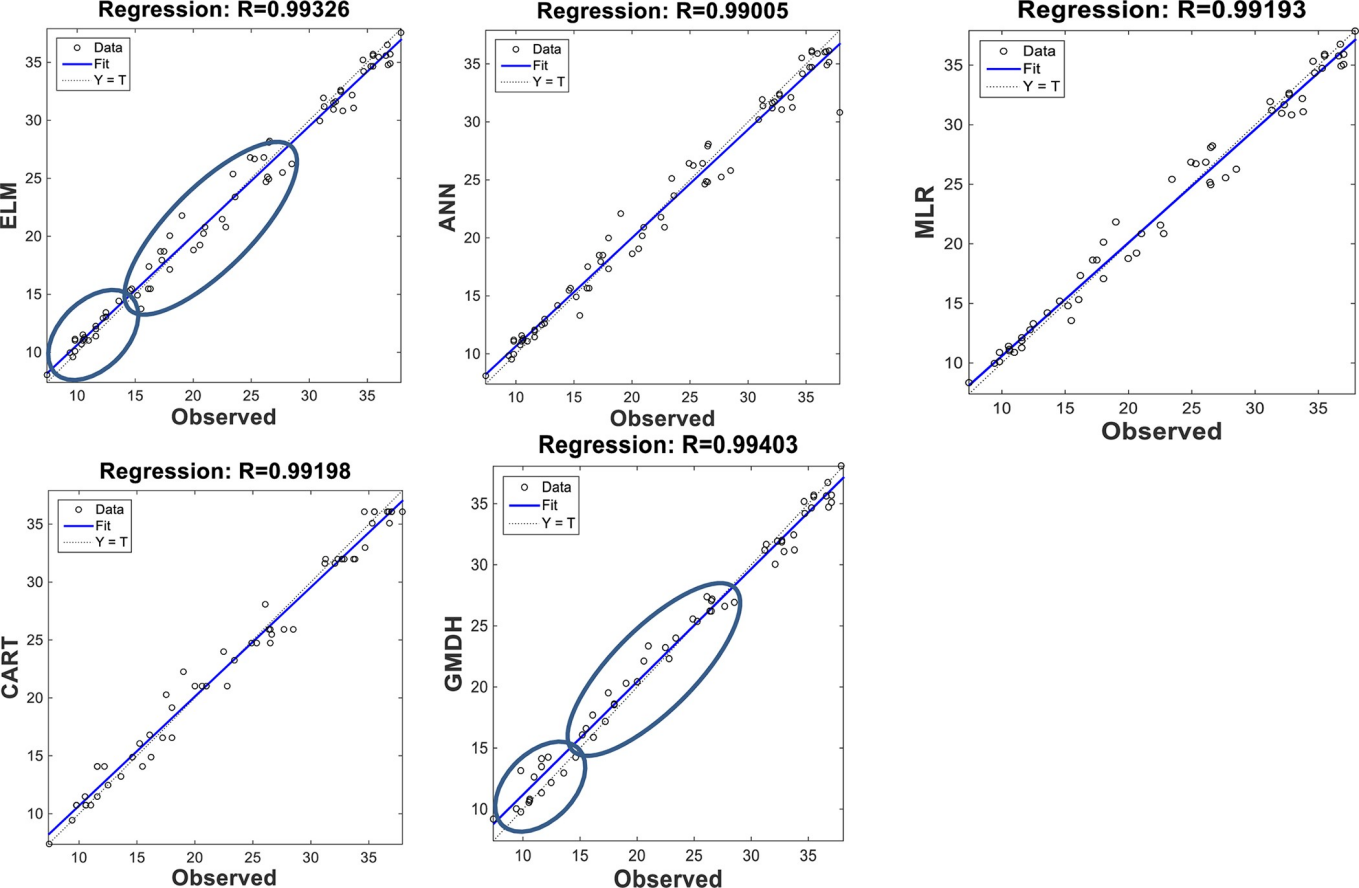
The scatterplots of the optimal ELM, ANN, CART, GMDH, and MLR models in predicting soil temperatures at 10 cm depth.

**Fig 14:**
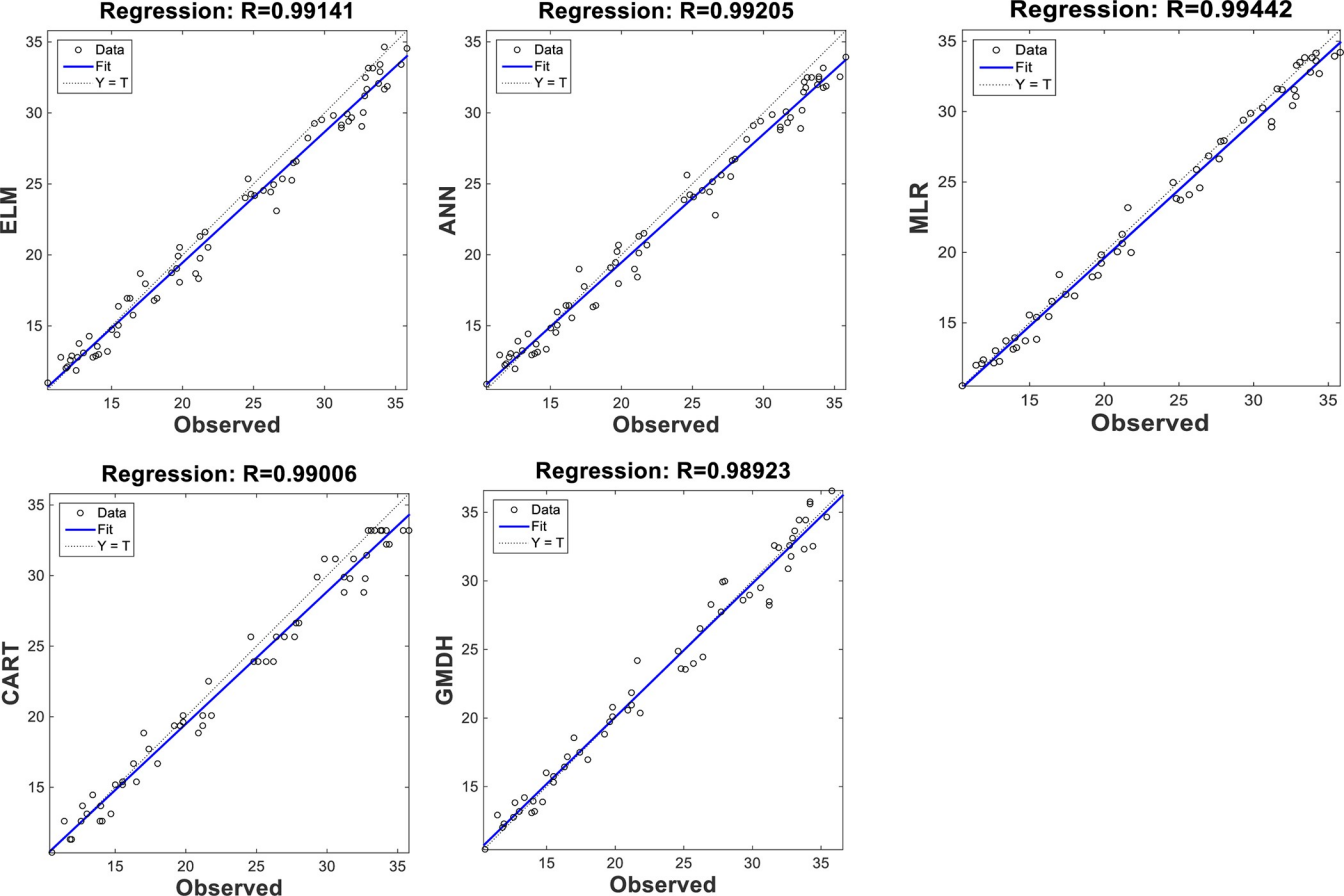
The scatterplots of the optimal ELM, ANN, CART, GMDH, and MLR models in predicting soil temperatures at 50 cm depth.

**Fig 15:**
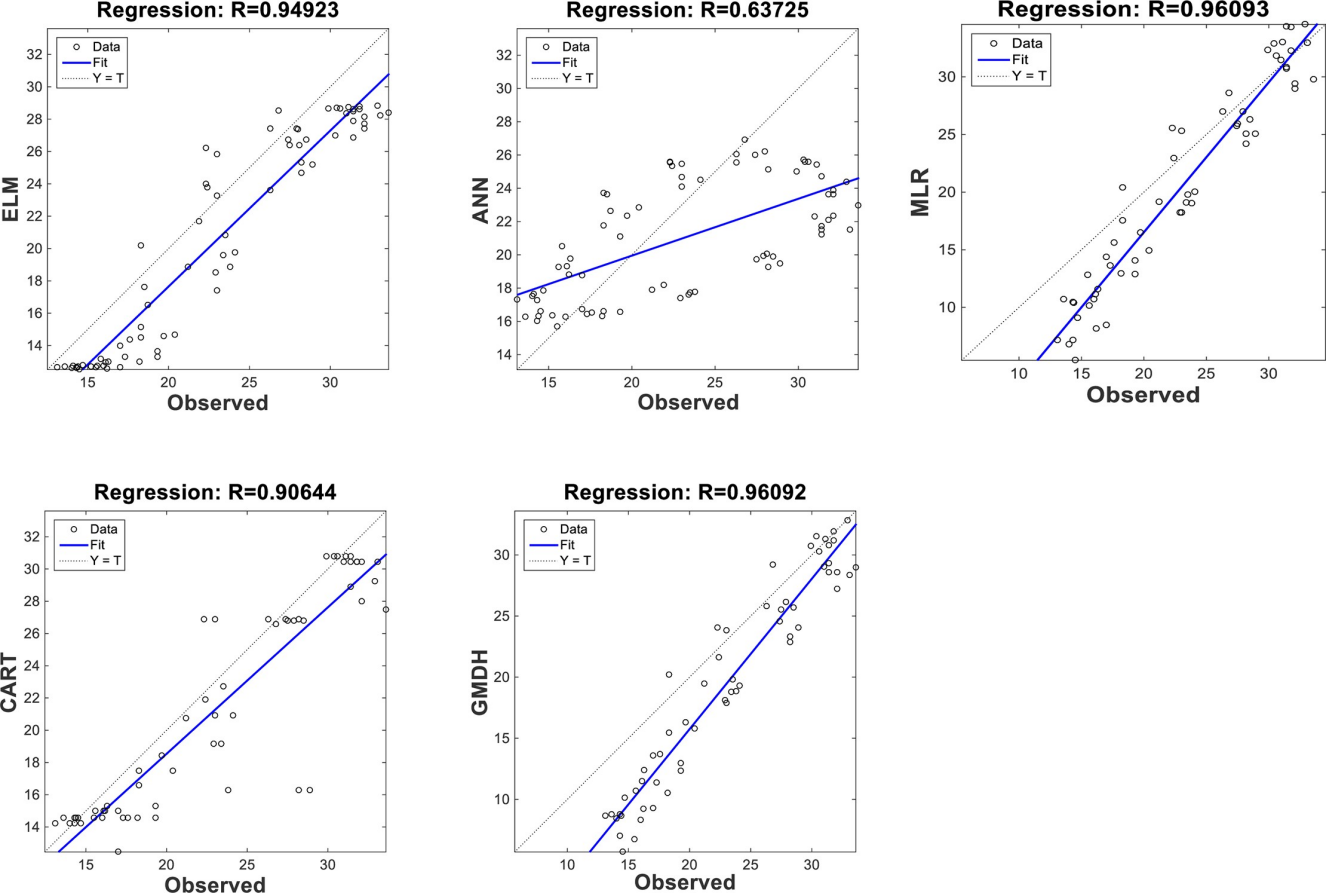
The scatterplots of the optimal ELM, ANN, CART, GMDH, and MLR models in predicting soil temperatures at 100 cm depth.

**Fig 16:**
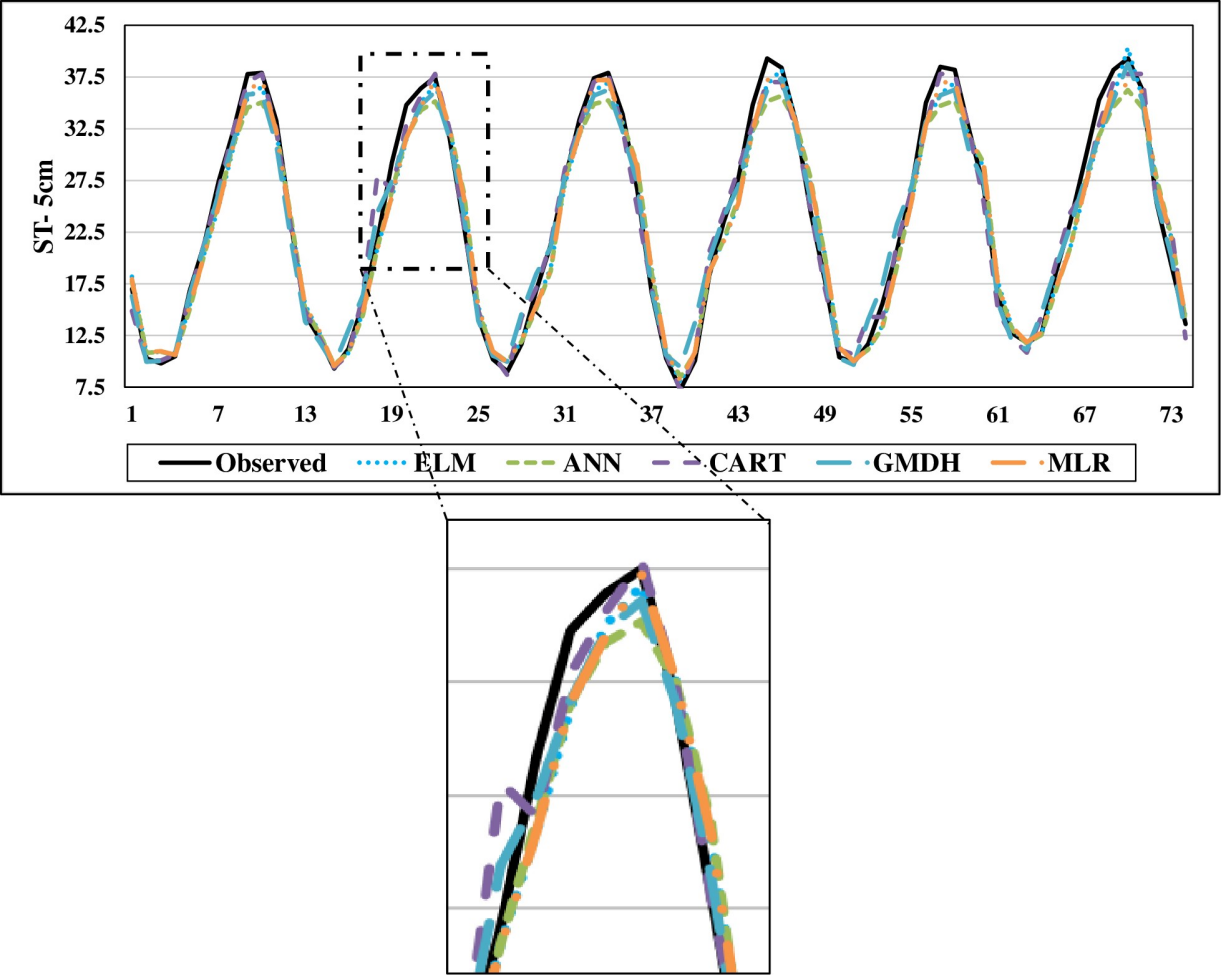
The comparison of the optimal ELM, ANN, CART, GMDH, and MLR models in predicting soil temperatures at 5 cm depth.

**Fig 17:**
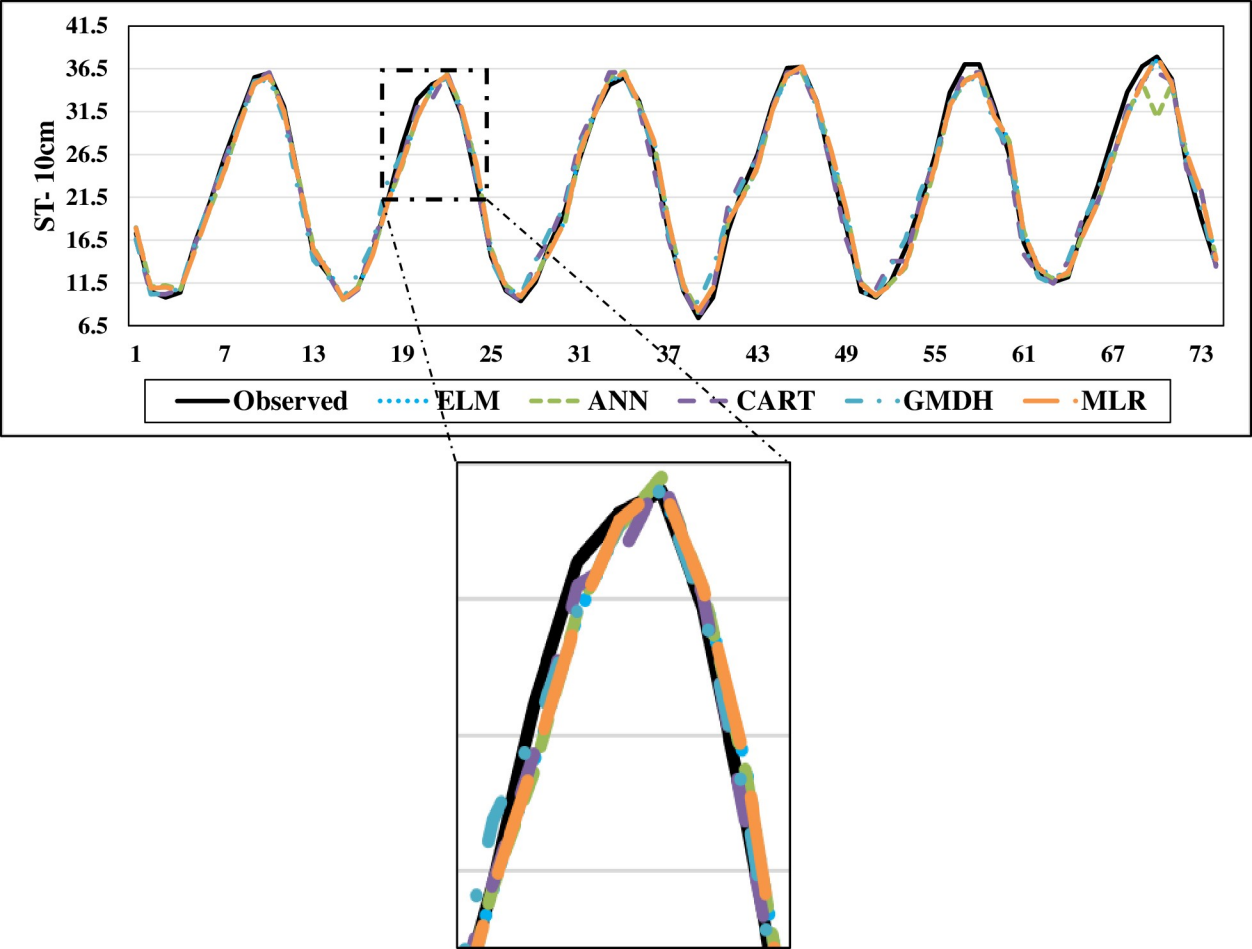
The comparison of the optimal ELM, ANN, CART, GMDH, and MLR models in predicting soil temperatures at 10 cm depth.

**Fig 18:**
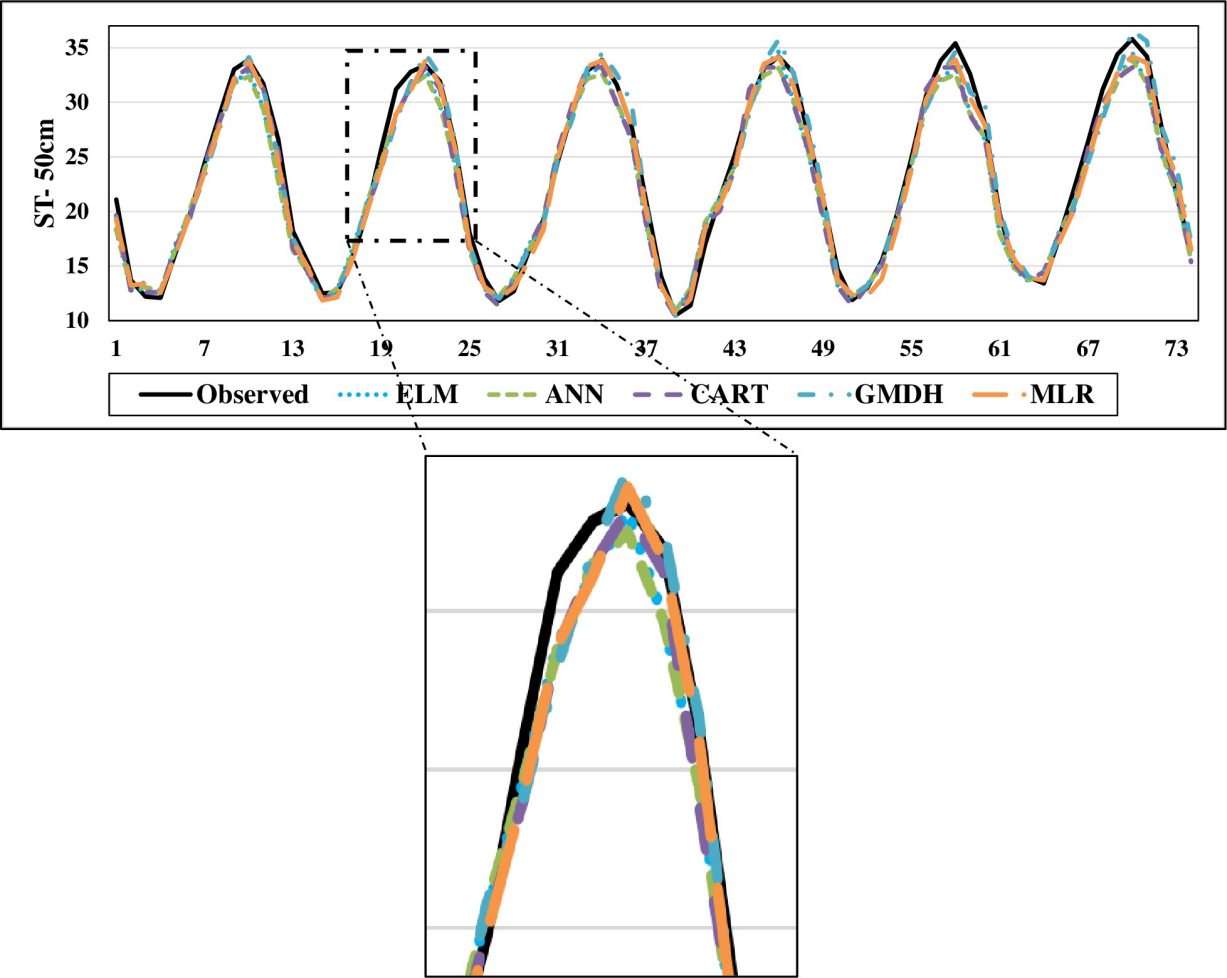
The comparison of the optimal ELM, ANN, CART, GMDH, and MLR models in predicting soil temperatures at 50 cm depth.

**Fig 19:**
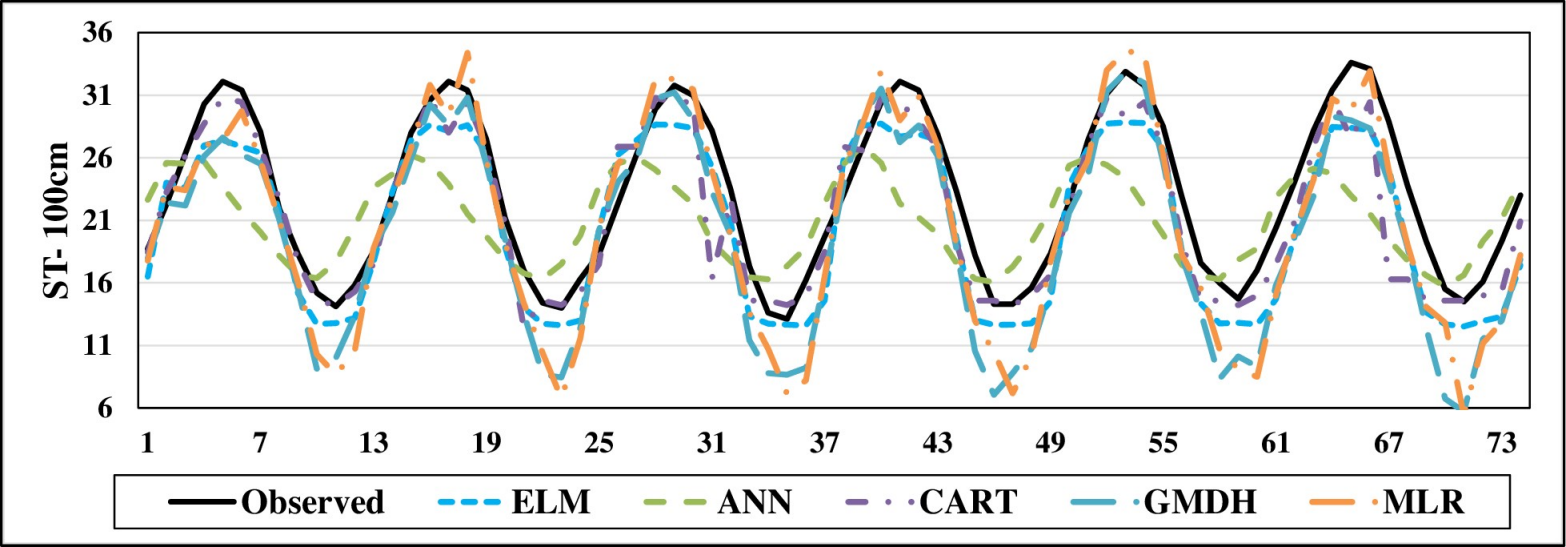
The comparison of the optimal ELM, ANN, CART, GMDH, and MLR models in predicting soil temperatures at 100 cm depth.

The scatterplots of the optimal models in estimating ST at various depths are shown in Figs 12–15. In case of soil temperatures at 5 cm depth (ST_5_) and at 10 cm depth (ST_10_), the ELM and GMDH generally have less scattered estimates than the CART and ANN models (Figs 12 and 13). The ELM better predicts low ST_5_ and ST_10_ (lower than 15 ^o^C) while the GMDH has better estimates middle values (between 15 and 30 ^o^C). Here, the main advantage of the ELM model compared to GMDH model is it uses only air temperature data while the latter model also requires relative humidity. In case of soil temperatures at 50 cm depth (ST_50_), the fit line of the GMDH model is closer to the exact line (Y(estimate) = T (target or observed)) which indicates that the estimates of the GMDH are closer to the observed values compared to ELM, ANN, CART, MLR model (Fig 14). All the optimal models use only temperature input. In case of soil temperatures at 100 cm depth (ST_100_), the ELM model is relatively better than the other models while the ANN model provides the worst results (Fig 15). Time variation of the models’ estimates and observed ST values are shown in Figs 16–19. All five methods could catch the general trend of the ST values at three depths while for the depth of 100 cm, considerable under- and over-estimations are observed for the all methods. The ANN seems to be inadequate in catching ST at 100 cm depth while the GMDH and MLR have considerable over- and underestimates at this depth. The main reason of this might be the low correlation between climatic inputs and ST at 100 cm depth. The other may be the different behavior of ST at 100 depth as also observed from Fig 2.

In overall, the ELM provides better accuracy than the ANN, CART, GMDH and MLR in estimating soil temperature at different multiple depths. This result is in accordance with the study of Feng et al. [19] in which ELM was applied in estimating soil temperature at the depths of 2, 5, 10 and 20 cm and compared with GRNN, BPNN and RF models. Better estimates were obtained from ELM compared to other models.

## Conclusion

The abilities of four machine learning methods, ELM, ANN, CART and GMDH in estimating soil temperature at different depths were compared utilizing various combinations of climatic variables as inputs and results were compared with MLR model. The following conclusions can be reached from application results:

- It was found that the models’ accuracies generally decrease by increase in soil depth.
- Soil temperatures at 5, 10 and 50 cm depths could be successfully predicted using only air temperature data as input. In prediction of ST at 100 cm depth, however, solar radiation and wind speed information are also needed.
- ELM method generally provided superior accuracy to the other methods in predicting monthly soil temperatures at various depths.
- ELM can be used in real soil temperature forecasting which carries importance for agricultural decision systems.

In the current research, models were tested by data from only one site. In future studies, the models may be tested using more data from other sites and ELM may be compared with other machine learning methods such as neuro-fuzzy, fuzzy-genetic, neuro-genetic.
